# N-acetyl cysteine and mushroom *Agaricus sylvaticus* supplementation decreased parasitaemia and pulmonary oxidative stress in a mice model of malaria

**DOI:** 10.1186/s12936-015-0717-0

**Published:** 2015-05-15

**Authors:** Bruno A. Quadros Gomes, Lucio F. D. da Silva, Antonio R. Quadros Gomes, Danilo R. Moreira, Maria Fani Dolabela, Rogério S. Santos, Michael D. Green, Eliete P. Carvalho, Sandro Percário

**Affiliations:** Oxidative Stress Research Laboratory, Institute of Biological Sciences, Federal University of Pará, Belém, Pará Brazil; Institute of Health Sciences, Federal University of Pará, Belém, Pará Brazil; Division of Parasitic Diseases and Malaria, US Centers for Disease Control and Prevention, 1600 Clifton Rd. NE, Mailstop G49, Atlanta, GA USA

**Keywords:** Antioxidants, Oxidative stress, *Agaricus sylvaticus*, N-acetyl cysteine, Nitric oxide, *Plasmodium berghei*, Malaria

## Abstract

**Background:**

Malaria infection can cause high oxidative stress, which could lead to the development of severe forms of malaria, such as pulmonary malaria. In recent years, the role of reactive oxygen species in the pathogenesis of the disease has been discussed, as well as the potential benefit of antioxidants supplementation. The aim of this study was to investigate the effects of N-acetyl cysteine (NAC) or mushroom *Agaricus sylvaticus* supplementation on the pulmonary oxidative changes in an experimental model of malaria caused by *Plasmodium berghei* strain ANKA.

**Methods:**

Swiss male mice were infected with *P. berghei* and treated with NAC or AS. Samples of lung tissue and whole blood were collected after one, three, five, seven or ten days of infection for the assessment of thiobarbituric acid reactive substances (TBARS), trolox equivalent antioxidant capacity (TEAC), nitrites and nitrates (NN) and to assess the degree of parasitaemia.

**Results:**

Although parasitaemia increased progressively with the evolution of the disease in all infected groups, there was a significant decrease from the seventh to the tenth day of infection in both antioxidant-supplemented groups. Results showed significant higher levels of TEAC in both supplemented groups, the highest occurring in the group supplemented with *A. sylvaticus*. In parallel, TBARS showed similar levels among all groups, while levels of NN were higher in animals supplemented with NAC in relation to the positive control groups and *A. sylvaticus*, whose levels were similar to the negative control group.

**Conclusion:**

Oxidative stress arising from plasmodial infection was attenuated by supplementation of both antioxidants, but *A. sylvaticus* proved to be more effective and has the potential to become an important tool in the adjuvant therapy of malaria.

**Electronic supplementary material:**

The online version of this article (doi:10.1186/s12936-015-0717-0) contains supplementary material, which is available to authorized users.

## Background

Malaria is one of the most prevalent human infections and a huge health, economic and social problem for more than 40 % of the world’s population [1–3]. It is estimated approximately 130 million cases occur each year, resulting in 315,000 to 689,000 deaths [1], of which 90 % occur in sub-Saharan Africa, mainly in children under five years of age [1, 2, 4]. Two-thirds of the remainder are concentrated in countries such as India, Pakistan, Myanmar, Papua New Guinea, and Brazil [1, 5]. It has been shown that the severity of the disease is mostly related to oxidative changes caused during *Plasmodium* infection. These changes occur when reactive oxygen species (ROS) produced by the infected red blood cell (RBC) cause imbalances of antioxidant defense mechanisms in the host. The resulting intravascular oxidative stress on endothelial cells contributes to malarial anaemia and the onset of severe forms of the disease [6–9].

In this context, when antioxidant protection mechanisms become unbalanced, physiological changes may occur, resulting in disease outcomes and accelerated aging. However, the use of antioxidant supplements may help reduce oxidative damage and to prevent disease evolution [8, 10–12]. Hence, it is possible that supplementation with antioxidant-rich sources can exert a preventive role against malaria development and become a protective strategy to exposed individuals, especially in endemic areas.

Among several sources of antioxidants that can carry out this role, two are particularly interesting: N-acetyl cysteine (NAC), which is a precursor of the hepatic synthesis of reduced glutathione (GSH) and mushrooms of the genus *Agaricus,* recently identified as bearers of high total antioxidant capacity [13, 14]. A more detailed description of the medicinal and antioxidant properties of this unique mushroom can be found at the Additional file 1.

### Pulmonary malaria

With regard to disease severity, pulmonary distress resulting from *Plasmodium falciparum* infection are reported in 3-10 % of cases, with variable clinical manifestations, ranging from discrete, related to the upper airways, to more severe complications, with severe hypoxia, pulmonary oedema and death. Acute pulmonary oedema is expected in approximately one-third of fatal cases of falciparum malaria among adults, with case fatality rates close to 70 % [15].

In children, respiratory disorder is a response to metabolic acidosis, predominantly due to an increase in the production of lactic acid and microvascular obstruction in the presence of anaemia. In addition, rigid erythrocytes can exacerbate microvascular obstruction and further impair blood flow, leading to the development of severe malaria in the acute phase of the disease [16].

Although the participation of lungs as one of the main components involved in the severity of malaria has been well documented [15, 17, 18], knowledge about this pathogenesis is still limited, despite being clinically characterized as severe and often lethal. It is known that the initial clinical presentation is acute respiratory distress syndrome, accompanied, in the more severe forms of the disease, by pulmonary oedema. The mechanisms responsible for triggering this complication seem to precede the increase in alveolar capillaries permeability, leading to lung fluid accumulation [17].

Nevertheless, the physiopathogenic mechanisms of pulmonary complications in malaria are not yet well known, and there are conflicting suggestions for the phenomenon. In fact, some researchers believe that pulmonary oedema is due to septic shock secondary to co-bacterial infection [17, 19]. On the other hand, others suggest that substances derived from parasitic infection, such as glycophosphatidylinositols and digestive vacuoles may contribute to the vascular changes that lead to pulmonary malaria [20]. Also, other researchers have described the occurrence of ischemia-reperfusion syndrome and the subsequent development of oxidative stress to be the result of the cyto-adherence characteristic of *P. falciparum* infection [14, 21, 22].

Hence, the primary objective of this study was to investigate and compare the potential benefits of antioxidant supplementation with mushroom *Agaricus sylvaticus* and NAC on lung oxidative changes and their impact over parasitaemia in an experimental model of malaria caused by *Plasmodium berghei* in mice. Both objectives were fully achieved.

## Methods

Two-hundred male Swiss mice (*Mus musculus*), young adults (25–35 g), from the Evandro Chagas Institute (Belem, PA, Brazil) were randomly divided into four groups, further divided in five sub-groups each (N = 10 each), according to time of animal’s euthanasia (one, three, five, seven or ten days after inoculation), and samples of lung tissue and blood were collected for the evaluation of oxidative stress markers, antioxidant defenses and degree of parasitaemia, as follows:

- Negative control groups (NC); N = 10 for each sub-group): animals that were inoculated with non-infected erythrocytes and received physiological saline solution (0.85 %; 0.4 mL/kg body weight, ip) before the study period. In addition, 10 μl of sterile distilled water per 25 g of body weight (gavage) was administered two hours before inoculation and daily, until the day of animals’ euthanasia.
- Positive control groups (PC); N = 10 for each sub-group): animals were inoculated with *P. berghei*-infected erythrocytes and received 10 μl of sterile distilled water per 25 g of body weight (gavage) two hours prior to the inoculation of *P. berghei* and daily, until the day of animals’ euthanasia.
- N-acetyl cysteine groups (NACG); N = 10 for each subgroup): animals were inoculated with *P. berghei* in the same way that groups PC and treated with NAC, as described below, until the day of animals’ euthanasia.
- Agaricus sylvaticus groups (ASG); N = 10 for each sub-group): animals were inoculated with *P. berghei* in the same way that groups PC and simultaneously treated with mushroom *Agaricus sylvaticus,* as described below, until the day of animals’ euthanasia.

All animals were assigned into sub-groups by simple randomization using a sub-group sequence generated after sortition [23] and were maintained in a vivarium at the Federal University of Pará (UFPA, Belém, PA, Brazil) in polystyrene cages containing five animals each, kept under 12 h light/dark cycles, controlled temperature (25 °C), and received rodent chow (Labina™, Presence, Brazil) and tap water *ad libitum* for one, three, five, seven or ten days after infection of animals for each sub-group and, at the end of each period, animals were submitted to heparin administration (heparin sulfate 100 IU, ip), anesthetized with 50 μL of Ketamine (5 %)-Xylazine (2 %), sample collection, and underwent euthanasia by hypovolemia after exsanguination. Absolutely all efforts were made to minimize suffering to animals.

After thoracotomy, blood samples were obtained by cardiac puncture of the right ventricle and both lungs were removed. The project followed the international guidelines for research with experimental animals and procedures were reviewed and approved by the Ethics Committee in Research with Experimental Animals of the Federal University of Pará - CEPAE/UFPA (Report No. MED014/2008).

### Features of the animal model

The use of Swiss mice as model for malaria infection is widely used and presents the same pattern of infection progression and basic features of lung malaria as other mice species. As described by Sadavongvivad and Aviado [24], the main histopathological lung findings in *P. berghei*-infected mice are the presence of inflammatory reaction around the alveoli and intra-alveolar haemorrhages. Moreover, the presence of large alveolar oedema is a common finding, often causing over 40 % increases in lung-to-body weight ratio [25, 26]. Additionally, the infiltration of macrophages and lymphocytes are observed as the infection progresses and are responsible for septal thickening [27, 28], as well as the presence of cytoadherence of mononuclear cells to pulmonary vessels [28]. Taken together, the histopathological features described are similar to those displayed in severe malaria human cases [15].

### Malaria induction

Mice were kept in the vivarium for two weeks and underwent clinical examination prior malaria induction through intraperitoneal inoculation of 10^6^ erythrocytes infected with *P. berghei* ANKA (in 0.2 mL of sterile saline solution). The strain of *P. berghei* was supplied by the Neurochemistry Laboratory of the Federal University of Pará - UFPA and replicated in Swiss mice by three times before being used in animals of this study.

### NAC and *Agaricus sylvaticus* administration

#### NAC

A 50 mg/mL aqueous solution of N-acetyl-L-cysteine (Fluimicil; Zambom Pharma, Italy) was prepared and administered to the animals (0.4 mL/kg body weight; gavage). Ten μl of NAC solution per 25 g of body weight was administered two hours before inoculation of *P. berghei* and daily until the day of animals’ euthanasia.

#### *Agaricus sylvaticus*

After owner’s written consent, fresh mushrooms were harvested in a private land at the city of Tapirai (Sao Paulo, Brazil; coordinates: 23°54’10.4” S, 47°31’25.0” W) and washed and brushed under tap water to remove dirt. It was identified as *A. sylvaticus* by Dr D Pegler (Kew Botanical Gardens, UK) and is not an endangered or protected species. Next, mushrooms were quartered and submitted to oven dehydration (60 °C, 12 h); the liquid suspension was prepared by steeping 1 g of powdered dried mushroom (80 meshes) in 100 mL of hot water (60 °C), which was then double-filtered with Millipore 0.45 and 0.25 μm. The resulting aqueous solution was administered to animals (0.2 mL/kg of body weight; gavage) two hours before inoculation of *P. berghei* and daily until the day of animals’ euthanasia.

### Sample obtaining and processing

Samples of lungs were collected for the evaluation of total antioxidant capacity and markers of oxidative stress. Lungs were exposed by thoracotomy and perfused with PBS to wash out the blood trapped inside. The tissue was weighed and added to PBS in the ratio of 1:10 (m:v). The homogenization process was performed in an ultrasonic cell disruptor (Thornton, Indaiatuba, Brazil; D Cel). During the process, the glass beaker containing the material was kept on ice to prevent sample damage. The homogenate was centrifuged at 175 x *g* (15 min) and the supernatant collected and stored in a freezer at −20 °C until analysed.

### Determination of parasitaemia

*Plasmodium berghei*-infected RBC were counted on blood smears obtained by puncture of the caudal vein of animals on the day of euthanasia (one, three, five, seven and ten days of infection). After drying at room temperature, the smear was fixed with methanol for 2 min and stained with Giemsa for 10 min. Subsequently, slides were washed in tap water and, after drying, RBC were counted in an optical microscope (Olympus, CX2) with 100x magnification (see Additional file 2).

### Technical procedure

Laboratory measurements of Trolox equivalent antioxidant capacity (TEAC), thiobarbituric acid reactive substances (TBARS) and nitrites and nitrates (NN) were performed in duplicate on lung tissue samples. Internal controls and standards were inserted in each batch for the quality assurance of determinations.

### Determination of Trolox equivalent antioxidant capacity (TEAC)

Trolox (6-hydroxy-2,5,7,8-tetramethylchromane-2-carboxylic acid; Sigma-Aldrich 23881–3) is a powerful antioxidant water-soluble analogue of vitamin E. The method proposed by Miller et al. [29] modified by Re et al. [30] was followed, a colorimetric technique based on the reaction between ABTS (2,2’-Azino-bis-3-ethylbenzothiazoline-6-sulphonic acid; Sigma-Aldrich; 1888) with ammonium persulphate potassium (K_2_S_2_O_8_; Sigma-Aldrich; 60490), producing the radical cation ABTS^●^+, chromophore of green/blue colour. The addition of antioxidants to ABTS^●^ + reduces it again to ABTS, on a scale dependent on antioxidant capacity, concentration of antioxidants and duration of the reaction. This can be measured by spectrophotometry by observing the change in absorbance read at 734 nm for 5 min (Fento, Sao Paulo, Brazil; 800 XI). Finally, the total antioxidant activity of the sample is calculated as its relationship with the reactivity of the Trolox as standard, through the implementation of standard curve under the same conditions.

### Determination of thiobarbituric acid reactive substances (TBARS)

TBARS is a method that evaluates lipid peroxidation and was used as an indicator of oxidative stress. This technique is based on the reaction of malondialdehyde (MDA), among other substances, with thiobarbituric acid (TBA) (Sigma-Aldrich T5500), in low pH and high temperature, yielding MDA-TBA complex of pink colour, and absorbance peak at 535 nm.

The technical procedure was performed according to the protocol proposed by Khon and Liversedge [31], adapted by Percario et al. [32]. In brief: initial TBA solution (10 nM) was prepared in phosphate monobasic potassium (KH_2_PO_4_ 75 mM; Synth; 35,210) adjusted to pH 2.5 with acetic acid. Two-hundred and fifty μL of sample was added to 500 μL of TBA solution, mixed and placed in a water bath (95 °C x 60 min); after cooling at room temperature, 2.0 ml of 1-butanol was added, vortex mixed and subsequently centrifuged (175 × *g* × 15 min); 1.0 ml of the supernatant was collected and read at 535 nm (Fento, São Paulo, Brazil; 800 XI). 1,1,3,3, tetraethoxypropane (Sigma-Aldrich; T9889) was used for the implementation of the standard curve.

### Nitrites and nitrates (NN)

Much of nitric oxide (NO) released into the bloodstream is swept by haemoglobin in erythrocytes or converted to nitrite (NO_2_^●-^) in the presence of molecular oxygen. Nitrite reacts with oxyhaemoglobin, leading to the formation of nitrate (NO_3_^●-^) and methaemoglobin. Due to its stability, NO_2_^●-^ has been widely used to confirm the prior existence of NO [33]. The evaluation of this parameter was performed by means of spectrophotometry (Kit Total Nitrite/Nitrate, R & D Systems, KGE001). This technique is based on the quantitative determination of NO, involving the enzyme nitrate reductase, which converts nitrate to nitrite, followed by colorimetric detection of nitrite as a product of pink colour, produced by the Griess reaction and that absorbs visible light at 540 nm (PerkinElmer, Victor X3). Nitrite concentration was calculated based on the absorbance found in the nitrites standard curve.

### Statistical analysis

Sample size was calculated by the method proposed by Dell et al. [34]. The occurrence of discrepant values (*outliers*) was investigated through calculation of interquartile range, which calculates the difference between the third quartile (Q3) and the first quartile (Q1), called dj. Any value lower than Q1 - 3/2 dj or greater than Q3 + 3/2 dj, was considered as *outlier* and, therefore, removed from mathematical calculations.

Aiming at investigating the existence of statistically significant differences between the studied variables between the groups, two factors analysis of variance (ANOVA) was applied, when the assumption of normality and homoscedasticity was met, or the Mann–Whitney test, when the assumption of normality was not met, which occurred in the case of variable parasitaemia. The tests used to access the normality and homoscedasticity of the variables were Kolmogorov-Smirnov and Levene tests, respectively. When the null hypothesis between mean differences between the variables of the study groups was rejected, Tukey’s test was applied, and when a statistically significant difference between medians was detected, Dunn’s test was applied. In addition, within the same group the differences between the initial values (one day of infection) and late values (ten days of infection) were studied by the Student’s unpaired *t* test.

The existence of correlation between the variables was also analysed by Pearson’s correlation coefficient, considering all points obtained in all groups simultaneously and separately for each group studied. For the purposes of tests ANOVA and Mann–Whitney, statistical package SigmaStat version 3.5 was used, whereas for the calculation of correlations the statistical package SPSS version 17.0 was used. All statistical tests were applied considering the significance level of 5 % (*p* < 0.05).

## Results

**Fig. 1:**
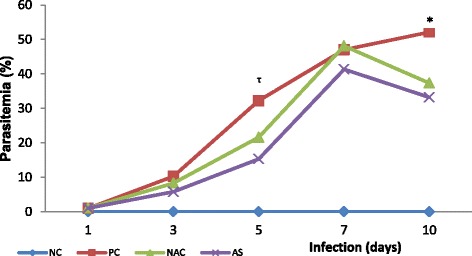
Parasitaemia progression in *Plasmodium berghei*-infected mice and supplemented with N-acetyl cysteine (NAC) or *Agaricus sylvaticus*. PC = animals infected with *P. berghei*, but not supplemented; NC = animals not infected and not supplemented. Group size: NC (1 day N = 10/10; 3 days N = 10/10; 5 days N = 10/10; 7 days N = 10/10; 10 days N = 10/10); PC (1 day N = 10/10; 3 days N = 10/10; 5 days N = 8^†¶^/10; 7 days N = 7^†^/10; 10 days N = 6^†^/10); NAC (1 day N = 10/10; 3 days N = 10/10; 5 days N = 10/10; 7 days N = 8^†^/10; 10 days N = 6^†^/10); AS (1 day N = 10/10; 3 days N = 10/10; 5 days N = 10/10; 7 days N = 8^†^/10; 10 days N = 5^†^/10). Discrepancies in group size were due to dead animals (^†^) or outliers (^¶^). * p = 0.0016 versus *Agaricus sylvaticus* (AS) and p = 0.0031 versus NAC, ^τ^ p < 0.0001 versus AS and p = 0.0034 versus NAC

Figure 1 shows the evolution of the parasitaemia of the positive control group and in the groups infected and supplemented with antioxidants. The parasitaemia increased in temporal scale for all three infected groups, being that on the tenth day of infection it was reduced in animals supplemented with NAC (37 ± 3 %, *p* = 0.0031) and *A. sylvaticus* (33 ± 4 %, *p* = 0.0016) when compared to group PC (52 ± 9 %).

**Fig. 2:**
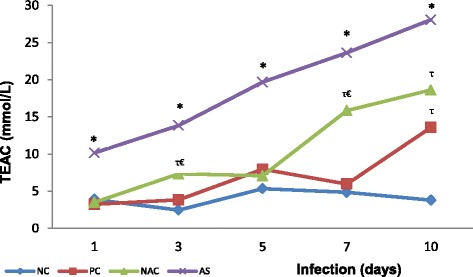
Pulmonary Trolox equivalent antioxidant capacity (TEAC) of *Plasmodium berghei-*infected mice in groups supplemented with N-acetyl cysteine (NAC) or *Agaricus sylvaticus* and control groups. PC = animals infected with *P. berghei*, but not supplemented; NC = animals not infected and not supplemented. Group size: NC (1 day N = 10/10; 3 days N = 10/10; 5 days N = 9^¶^/10; 7 days N = 10/10; 10 days N = 10/10); PC (1 day N = 10/10; 3 days N = 9^¶^/10; 5 days N = 9^†^/10; 7 days N = 6^†¶^/10; 10 days N = 6^†^/10); NAC (1 day N = 10/10; 3 days N = 8^¶^/10; 5 days N = 10/10; 7 days N = 8^†^/10; 10 days N = 6^†^/10); AS (1 day N = 10/10; 3 days N = 9^¶^/10; 5 days N = 10/10; 7 days N = 8^†^/10; 10 days N = 5^†^/10). Discrepancies in group size were due to dead animals (^†^) or outliers (^¶^).* p < 0.0001 versus PC, NAC, or NC. ^τ^ p < 0.01 versus NC. ^€^ p < 0.01 versus PC

In relation to the TEAC, all infected groups (PC, NACG and ASG) showed gradual elevation over time, with statistically higher values on the tenth day after infection, however, group ASG presented the highest elevation of total antioxidant capacity in relation to the other groups (4.0 ± 1.5 mmol/L for NC; 13.6 ± 4.5 mmol/L for PC; 18.6 ± 3.5 mmol/L for NACG; and, 28.1 ± 5.8 mmol/L for ASG; *p* < 0.0001) at the end of the study and throughout the period of infection (Fig. 2). In the same way, NACG showed higher antioxidant capacity if compared to the negative control group at three (7.3 ± 0.8 versus 2.5 ± 1.0 mmol/L, respectively; *p* < 0.01), seven (16.5 ± 5.0 versus 4.8 ± 0.6 mmol/L, respectively; *p* < 0.01) and ten days of infection (18.6 ± 3.5 versus 4.0 ± 1.5 mmol/L, respectively; *p* < 0.01) and also against the positive control group on the third (7.3 ± 0.8 versus 3.8 ± 0.8 mmol/L, respectively; *p* < 0.01) and seventh days (16.5 ± 5.0 versus 6.0 ± 0.5 mmol/L, respectively; *p* < 0.01).

**Fig. 3:**
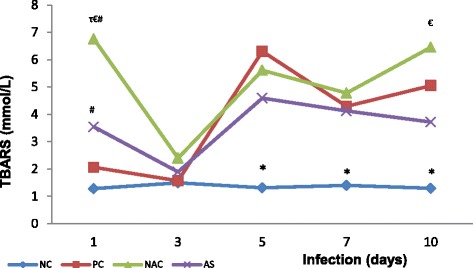
Pulmonary thiobarbituric acid reactive substances (TBARS) of *Plasmodium berghei-*infected mice in groups supplemented with N-acetyl cysteine (NAC) or *Agaricus sylvaticus* and control groups. PC = animals infected with *P. berghei*, but not supplemented; NC = animals not infected and not supplemented. Group size: NC (1 day N = 8^¶^/10; 3 days N = 10/10; 5 days N = 10/10; 7 days N = 10/10; 10 days N = 9^¶^/10); PC (1 day N = 10/10; 3 days N = 10/10; 5 days N = 9^†^/10; 7 days N = 7^†^/10; 10 days N = 6^†^/10); NAC (1 day N = 8^¶^/10; 3 days N = 8^¶^/10; 5 days N = 10/10; 7 days N = 8^†^/10; 10 days N = 6^†^/10); AS (1 day N = 10/10; 3 days N = 10/10; 5 days N = 10/10; 7 days N = 8^†^/10; 10 days N = 5^†^/10). Discrepancies in group size were due to dead animals (^†^) or outliers (^¶^).* p < 0.001 versus PC, NAC, or *Agaricus sylvaticus* (AS). ^τ^ p < 0.01 versus PC, ^€^ p < 0.01 versus AS, ^#^ p < 0.05 versus NC

In addition, in PC group there was an increase in TBARS levels with the progression of the disease from the first to the tenth day of infection (2.1 ± 1.2 mmol/L and 5.1 ± 0.9 mmol/L, respectively; *p* = 0.0002). Similarly, there was a significant increase of TBARS in the groups of supplemented animals when compared the tenth day of infection versus the third day for the same groups (6.5 ± 1.9 mmol/L and 2.4 ± 0.8 mmol/L, respectively; *p* = 0.0001 for NACG and 3.7 ± 1.2 mmol/L and 1.9 ± 0.7 mmol/L, respectively; *p* = 0.0020 for ASG). Moreover, the group of animals supplemented with NAC presented higher levels of TBARS in relation to the other groups on the first day of infection (6.8 ± 1.8 mmol/L for NACG; 3.5 ± 2.0 mmol/L for ASG; 2.1 ± 1.2 mmol/L for PC, and 1.3 ± 0.1 mmol/L for NC; *p* < 0.01). No significant differences were observed between NACG or ASG and positive control group for three, five, seven, or ten days of infection. However, ASG has presented lower levels of TBARS than NACG on the tenth day of infection (3.7 ± 1.2 mmol/L and 6.5 ± 1.9 mmol/L, respectively; *p* < 0.01; Fig. 3).

**Fig. 4:**
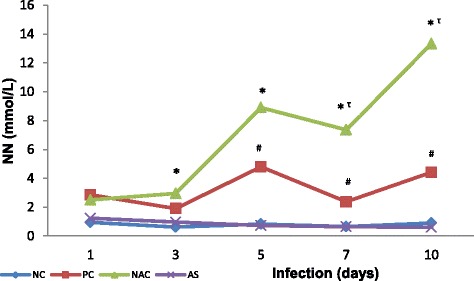
Pulmonary nitrites and nitrates (NN) of *Plasmodium berghei-*infected mice in groups supplemented with N-acetyl cysteine (NAC) or *Agaricus sylvaticus* and control groups. PC = animals infected with *P. berghei*, but not supplemented; NC = animals not infected and not supplemented. Group size: NC (1 day N = 9^¶^/10; 3 days N = 8^¶^/10; 5 days N = 9^¶^/10; 7 days N = 8^¶^/10; 10 days N = 9^¶^/10); PC (1 day N = 9^¶^/10; 3 days N = 10/10; 5 days N = 9^†^/10; 7 days N = 7^†^/10; 10 days N = 6^†^/10); NAC (1 day N = 10/10; 3 days N = 8^¶^/10; 5 days N = 9^¶^/10; 7 days N = 6^†¶^/10; 10 days N = 6^†^/10); AS (1 day N = 10/10; 3 days N = 10/10; 5 days N = 9^¶^/10; 7 days N = 8^†^/10; 10 days N = 5^†^/10). Discrepancies in group size were due to dead animals (^†^) or outliers (^¶^). * p < 0.01 versus *Agaricus sylvaticus* (AS) or NC, ^τ^ p < 0.01 versus PC, ^#^ p < 0.05 versus NC or AS

Similarly, in relation to the levels of NN, NACG showed progressive increase with the time of infection since the first day (*p* = 0.0025), while group PC presented a less pronounced increase than the NACG and was statistically significant from the fifth day of infection (*p* < 0.05). Animals supplemented with NAC presented values of NN significantly elevated compared to ASG and NC (*p* < 0.01 and *p* < 0.01, respectively) from the third to the tenth day of the study, in addition to values higher than the PC group from the fifth to tenth day of infection (*p* < 0.05). On the other hand, animals supplemented with *A. sylvaticus* showed NN levels similar to those presented by the negative control group throughout the study period (Fig. 4; see Additional file 3).

### Correlation studies

The analysis of correlation between TBARS and NN showed weak positive correlation for groups PC and NACG (r^2^ = 0.0895, *p* = 0.0008 and r^2^ = 0.1598, *p* = 0.0174, respectively), however, for ASG this correlation was not significant (r^2^ = 0.0001 and *p* = 0.3328; see Additional file 4).

Additional file 5 show the correlation between TEAC and NN, demonstrating the existence of moderate positive correlation of highly significance for NACG (r^2^ = 0.3562, *p* = 0.0002). On the other hand, in ASG a weak negative correlation was found (r^2^ = −0.0394, *p* = 0.0120).

In relation to parasitaemia, there was a positive correlation with TEAC (see Additional file 6), NN (see Additional file 7) and TBARS (see Additional file 8), when considering all values obtained in all groups and sub-groups simultaneously (r^2^ = 0.2153, *p* < 00001; r^2^ = 0.0707, *p* = 0.0012, r^2^ = 0.1042, *p* = 0.0003, respectively), as well as for PC (r^2^ = 0.5273, *p* < 00001; r^2^ = 0.3068, *p =* 0.0474 and r^2^ = 0.5839, *p* < 0.0001, respectively). For NACG, a positive correlation between parasitaemia and NN (r^2^ = 0.3367, *p* = 0.0003) and between parasitaemia and TEAC (r^2^ = 0.6622, *p* < 0.0001) was also found. In ASG, positive correlations between parasitaemia and TEAC and parasitaemia and TBARS were found (r^2^ = 0.8242, *p* < 00001 and r^2^ = 0.4265, *p* = 0.0068, respectively), in addition to negative correlation between parasitaemia and NN (r^2^ = −0.4410, *p* = 0.0044).

## Discussion

Severe forms of malaria are generally accompanied by impairment of pulmonary tissue, initially involving the acute respiratory distress syndrome, accompanied, in the more severe forms of the disease, by acute pulmonary oedema [17, 35]. These changes may be the result of the cyto-adherence phenomenon, particularly peculiar to *P. falciparum*, likely involving ischemia and reperfusion syndrome, with consequent free radical production and oxidative stress [9, 21, 22]. Thus, the evaluation of the involvement of oxidative stress and its correlation with the parasitaemia in the severe forms of the disease, as well as the potential beneficial effect of the use of antioxidants, may provide a new approach to the treatment of disease, especially in children who live in endemic areas.

However, studies of the potential beneficial effects of supplementation with antioxidants performed so far presented complex and often conflicting results [12], creating a discouraging expectation for the practical application of these supplements in the adjuvant therapy of malaria. Nevertheless, the analysis of the effects of antioxidant supplements is very difficult and complex, as they elapse from direct actions of the supplements on target molecules, but also depend on a large number of mechanisms of indirect interaction, often related to multiple pathways of cellular signaling. The choice of antioxidant supplements employed in this study was based on the antioxidant properties they present. For example, NAC is an exogenous source of glutathione [36, 37], which is one of the most important endogenous antioxidant molecules, whereas mushroom *A. sylvaticus* is composed of various antioxidant molecules that, in combination, have one of the highest values of antioxidant capacity currently found in natural products [14]. It is responsible for several therapeutic and preventive effects reported in recent literature [11, 38–42]. Nevertheless, it was never used before against malaria. Additionally, NAC is commercially used for the treatment of lung disorders whereas AS is commonly used as an antioxidant supplement in Brazil. In both cases a decision was made to use the average dose employed for humans, i.e. 20 mg/kg of body weight for NAC and 2 mg/kg of body weight for AS.

The analysis of the present results showed that, as expected, parasitaemia progresses in temporal scale in the positive control group (Fig. 1), which is in agreement with the findings of other authors who have used similar rodent models of malaria [4, 43–45]. For NACG and ASG, there was similar behaviour to that of the positive control, however, in addition to presenting a lower progression rate than the positive control group, there was a significant decrease of parasitaemia from day 7 of infection, suggesting the parasite requires a pro-oxidant environment to develop and that antioxidant supplementation is unfavourable for the progression of this infection. This suggestion is strengthened by the finding of significant positive correlation between parasitaemia and TBARS - marker of oxidative stress - for the animals in the positive control group and that it is abolished in groups of animals supplemented with antioxidants (see Additional file 8).

Moreover, animals treated with AS displayed an average of 43 % parasitemia reduction when compared with PC animals, whereas NAC animals displayed an average of 29 % reduction. Despite no significant differences to animals’ survival in the short term (see Additional file 9), this important decrease in parasitemia undoubtedly reflects the effects of both supplements over parasite development. Eventually, this might lead to a positive reflex over time. Alternatively, the lack of differences in the survival rate could be a result of the relatively small number of animals in each group, and large scale studies might show better results.

Likewise, parasitaemia total antioxidant capacity also increases during infection for all groups infected with *Plasmodium* (PC, NACG and ASG; Fig. 2), which suggests mobilization of endogenous antioxidant molecules by the host as a probable mechanism of defence against oxidative stress generated during malarial infection. In fact, in an attempt to prevent oxidative stress, pulmonary micro-environment seems to rely on a strong antioxidant defence, in particular in the course of respiratory deficiency syndromes. Substances potentially responsible for this process are: proteins A1, A2 and B of alveolar surfactant [46], the enzymes catalase [47] and superoxide dismutase [48, 49], in addition to GSH [50].

Nevertheless, in antioxidant-supplemented groups, TEAC was even higher than in the control group, being found in ASG values significantly higher as compared to the other groups throughout the period of infection (Fig. 2). In addition, NACG showed higher antioxidant capacity if compared to the negative control group at three, seven and ten days of infection (*p* < 0.01) and also against positive control group on the third and seventh days (*p* < 0.01).

The increase of TEAC on the tenth day of infection in groups of animals supplemented with antioxidants occurred simultaneously with the decrease of parasitaemia during this period, particularly in animals supplemented with *A. sylvaticus* (*p <* 0.01, Fig. 2; see Additional file 6), which may suggest the existence of a particular level of antioxidant defence from which parasitaemia does not progress, likely because once the body creates an antioxidant environment unfavourable to the development of the parasite, disease may progress to resolution.

The fact is that in all the infected groups individually, there was a positive and highly significant correlation between parasitaemia and TEAC (see Additional file 6). This strongly suggests that as parasitaemia increases, the mobilization of endogenous antioxidant defences by pulmonary cells occurs, increasing total antioxidant capacity in an attempt to combat the infection, and that supplementation with antioxidants intensify this response even more.

Moreover, the presence of oxidative stress was assessed by TBARS levels, which increased with the progression of the disease for all infected groups (Fig. 3), suggesting that oxidative stress may result from infection, and increase significantly, even precociously. This oxidative stress may be a mechanism of host’s natural defence, depending on the activation of phagocytes (macrophages and neutrophils), which generate large amounts of ROS, causing an imbalance between the formation of oxidant and antioxidant molecules. This can lead to endothelial cell apoptosis and the worsening of the disease, as suggested by Hemmer et al. [51] and Trivedi et al. [52]. However, at least apparently, supplementation did not promote the reduction of TBARS levels in comparison to the PC group. However, when compared with each other, the groups supplemented with antioxidants showed significant differences on the tenth day of infection, with ASG displaying lower values of TBARS than NACG, reinforcing the suggestion that mushroom *Agaricus sylvaticus* supplementation had the ability to generate a more pronounced antioxidant defence in animals.

In relation to NN levels (Fig. 4), the increase found in positive control group animals suggests the involvement of nitric oxide synthesis due to malarial infection, a fact reinforced by the existence of significant positive correlation between parasitaemia and NN, as well as between TBARS and NN for the positive control group. These results are in agreement with several studies [53–56] and corroborate with the suggestion that NO synthesis is a protective factor against the development of severe malaria [57, 58]. Additionally, due to the simultaneous increase of free radicals, as suggested by TBARS levels found, there is a great possibility of radical peroxynitrite (ONOO^●-^) formation from the reaction between NO and superoxide radical (O_2_^●-^) probably produced by intensification of inflammatory process or resulting from ischemia and reperfusion, as a result of cyto-adherence of parasitized erythrocytes to pulmonary capillaries’ endothelium. Furthermore, there is the possibility of NO depletion due to intravascular haemolysis, which also lead to pulmonary hypertension, as has been studied by Janka et al. [59].

On the other hand, animals supplemented with NAC showed values of NN significantly higher than group AS (*p* <0.01) from the third day of infection, as well as in relation to the positive control group since the fifth day of infection (*p* <0.01). This may be the result of the ability of NAC to induce NO synthesis [60–62]. In parallel, there was a moderate positive and highly significant correlation between parasitaemia and NN in this group of animals (r^2^ = 0.3367 and *p* = 0.0003) whereas ASG showed a negative correlation (r = −0.4410 and *p* = 0.0044; see Additional file 7). In addition, in NACG it was also found positive and significant correlation between TBARS and NN (r^2^ = 0.1598 and *p* = 0.0174; see Additional file 4). In the correlation study between TEAC and NN, a moderate positive and highly significant correlation (r^2^ = 0.3562, *p* = 0.0002; see Additional file 5) between the parameters was observed for NACG.

These data reinforce the suggestion that NAC is related to the induction of NO and that it is associated with an increase in the total antioxidant capacity. In this sense, NAC can prevent or reverse haem’s harmful effects and act against oxidative damage to erythrocytes’ cytoskeleton [63], as well as act as a reducing agent due to the presence of the thiol group in the side chain of the molecule, and that it facilitates the expression of inducible nitric oxide synthase (iNOS) by IL-1β in vascular cells of rats [59, 64–66].

In children with severe malaria, NAC increases the normalization rate of plasma lactate by a mechanism dependent on TNF, possibly by increasing the deformity of RBC or by an increase in GSH [67]. Therefore, reducing agents, such as NAC, may have a therapeutic role in the complications of malaria and can reduce microvascular obstruction to the blood flow. Indeed, recent studies show that the increase in NO production has shown beneficial effects against malarial infection, but, although there is still controversy in this regard, these effects may be due to cyto-adherence inhibition through the reduction of the expression of intercellular adhesion molecule 1 (ICAM-1), vascular cell adhesion molecule 1 (VCAM-1), and E-selectin, which are involved in cyto-adherence and microvascular sequestration of parasitized RBC and that impairs the production of tumour necrosis factor alpha (TNF) by macrophages [68].

Nevertheless, if the induction of NO synthesis is exacerbated, the increase in NO levels, in association with the increase of ROS, can contribute to the severity of the disease at the pulmonary level. In this sense, Zhu et al. [69] observed that *P. falciparum* glycosylphosphatidylinositols induce the expression of iNOS and can positively regulate the expression of adhesion molecules by the vascular endothelium, probably implicated in parasite internalization to RBC. Anstey et al. [70], studying adults with vivax malaria and not complicated falciparum malaria, observed a reduction of the volume of pulmonary capillaries due to sequestration of parasitized red blood cells in the alveolar capillaries and of leukocytes in pulmonary vessels, as well as that the injuries caused to alveolar capillaries are due to inflammatory response to destroy the parasite or by reperfusion injury.

Contrary to NACG, the animals supplemented with *Agaricus sylvaticus* curiously showed levels of NN similar to the negative control group (Fig. 4; see Additional file 3), which suggests that during infection *A. sylvaticus* acts by inhibiting NO synthesis, by means of an intrinsic mechanism, or through direct NO scavenging, favouring the creation of an antioxidant environment within erythrocytes unfavourable to parasite internalization and further development, and probably decreasing apoptosis, contributing to cell integrity of pulmonary capillaries.

In ASG, a negative and significant correlation between TEAC and NN was found (r^2^ = −0.0394 and *p* = 0.0120; see Additional file 5), which reinforces the idea that *A. sylvaticus* may be involved in the inhibition of the synthesis and/or decrease in pulmonary NO concentration, with consequent increase of total antioxidant capacity. The same behaviour was found for the correlation between parasitaemia and NN (r^2^ = −0.4410 and *p* = 0.0044; see Additional file 7), strongly suggesting that *A. sylvaticus* plays an inhibitor role upon pulmonary NO synthesis and/or on the decrease of NO concentration as parasitaemia progresses, probably through an antioxidant defence pathway against infection. This suggestion is in agreement with Dimmeler et al. [71] who observed that TNF-α induced endothelial cells apoptosis was diminished after iNOS inhibition by N-monomethyl-L-arginine (L-NMMA). Furthermore, Pino et al. [72] observed that the adhesion of parasitized erythrocytes to human pulmonary endothelium may be regulated by TNF-α, cytokine that induces the expression of iNOS enzymes, suggesting that the inhibition of NO synthesis may protect pulmonary endothelial cells. Nevertheless, *A. sylvaticus* and NAC seem to act by two distinct mechanisms in order to fight the infection. On the one hand, NO synthesis inhibition by *A. sylvaticus* seems to be a little more relevant than the effects of NAC and probably exerts its effects in the longer term, by inhibiting infection-induced NO synthesis, keeping NO levels equivalent to baseline values and decreasing endothelial apoptosis and the internalization of the parasite in RBC. On the other hand, the induction of NO synthesis stimulated by NAC also presents some beneficial effect on parasite clearance and can improve microcirculatory flow, but seems to be dependent on high NO concentrations, which may culminate in the worsening of the disease, due to the production of other NO-derived molecules, such as ONOO^●-^. At the same time, NAC can act by scavenging ROS generated during infection and increasing total antioxidant capacity.

## Conclusion

Supplementation with both antioxidants promoted the reduction of parasitaemia and increase in antioxidant capacity, being both effects more pronounced in animals supplemented with *A. sylvaticus. Agaricus sylvaticus* and NAC possibly act by two distinct mechanisms in order to decrease plasmodial infection, as *A. sylvaticus* seems to act by inhibiting NO synthesis and increasing total antioxidant capacity, while NAC can act by inducing NO synthesis. Nevertheless, further studies are required to a better understanding about the precise mechanism by which antioxidants act upon evolution of parasitaemia and disease development, as well as its repercussions on the development of cerebral malaria, opening up new possibilities for the adjuvant therapy of this disease or other diseases involving oxidative stress.

## Additional files

Additional file 1: **Medicinal properties of mushroom** ***Agaricus sylvaticus*** **.** Presents the literature available about the medicinal properties of mushroom *Agaricus sylvaticus* and nutritional facts table of.

Additional file 2: **Number of red blood cells to be counted.** Contains a board of number of red blood cells to be counted from an initial parasitemia estimative.

Additional file 3: **Pulmonary Nitrites and Nitrates (NN) of** ***Plasmodium berghei-*** **infected mice in groups supplemented with N-Acetyl cysteine (NAC) or** ***Agaricus sylvaticus*** **(AS) and control groups, accordingly to duration of infection.** Presents mean ± standard deviation values of nitrites and nitrates for each group.

Additional file 4: **Correlation between Nitrites and nitrates (NN) and Thiobarbituric acid reactive substances (TBARS) in lung tissue of mice.** Present the correlation study charts of NN versus TBARS and consolidated charts of the variation of the average values of NN and TBARS in lung tissue of mice with the time of infection for each group.

Additional file 5: **Correlation between Nitrites and nitrates (NN) and Trolox equivalent antioxidant capacity (TEAC) of lung tissue of mice.** Present the correlation study charts of NN versus TEAC and consolidated charts of the variation of the average values of NN and TEAC in lung tissue of mice with the time of infection for each group.

Additional file 6: **Correlation between Parasitemia and Trolox equivalent antioxidant capacity (TEAC) of lung tissue of mice.** Present the correlation study charts of Parasitemia versus TEAC and consolidated charts of the variation of the average values of Parasitemia and TEAC in lung tissue of mice with the time of infection for each group.

Additional file 7: **Correlation between Parasitemia and Nitrites and nitrates (NN) of lung tissue of mice.** Present the correlation study charts of Parasitemia versus NN and consolidated charts of the variation of the average values of Parasitemia and NN in lung tissue of mice with the time of infection for each group.

Additional file 8: **Correlation between Parasitemia and Thiobarbituric acid reactive substances (TBARS) in lung tissue of mice.** Present the correlation study charts of Parasitemia versus TBARS and consolidated charts of the variation of the average values of Parasitemia and TBARS in lung tissue of mice with the time of infection for each group.

Additional file 9: **Survival rate of** ***Plasmodium berghei-*** **infected mice in groups supplemented with N-Acetyl cysteine (NAC) or** ***Agaricus sylvaticus*** **(AS) and control groups, accordingly to duration of infection.** Presents the survival rate chart for all groups.

## Abbreviations

ASG: *Agaricus sylvaticus* groups
ICAM-1: Intercellular adhesion molecule 1
iNOS: Inducible nitric oxide synthase
GSH: Reduced glutathione
MDA: Malondialdehyde
NAC: N-acetyl cysteine
NACG: N-acetyl cysteine groups
NC: Negative control groups
NO: Nitric oxide
NN: Nitrites and nitrates
L-NMMA: N-monomethyl-L-arginine
PC: Positive control groups
ROS: Reactive oxygen species
RBC: Red blood cell
TBA: Thiobarbituric acid
TBARS: Thiobarbituric acid reactive substances
TEAC: Trolox equivalent antioxidant capacity
TNF-α: Tumour necrosis factor alpha
VCAM-1: Vascular cell adhesion molecule 1

## References

[1] WHO. World Malaria Report 2014*.* World Health Organization, Geneva, 2014 [http://www.who.int/malaria/publications/world_malaria_report_2014/en/]

[2] Suh KN, Kain KC, Keystone JS (2004). Malaria. CMAJ.

[3] Potter SM, Mitchell AJ, Cowden WB, Sanni LA, Dinauer M, Haan JB (2005). Phagocyte-derived reactive oxygen species do not influence the progression of murine blood-stage malaria infections. Infect Immun.

[4] Reis PA, Comim CM, Hermani F, Silva B, Barichello T, Portella AC (2010). Cognitive dysfunction is sustained after rescue therapy in experimental cerebral malaria, and is reduced by additive antioxidant therapy. PLoS Pathog.

[5] Lou J, Lucas R, Grau GE (2001). Pathogenesis of cerebral malaria: recent experimental data and possible applications for humans. Clin Microbiol Rev.

[6] Omodeo-Salè F, Motti A, Basilico N, Parapini S, Olliaro P, Taramelli D (2003). Accelerated senescence of human erythrocytes cultured with *Plasmodium falciparum*. Blood.

[7] Tanneur V, Duranton C, Brand VB, Sandu CD, Akkaya C, Kasinathan RS (2006). Purinoceptors are involved in the induction of an osmolyte permeability in malaria-infected and oxidized human erythrocytes. FASEB J.

[8] Jaramillo M, Godbout M, Olivier M (2005). Hemozoin induces macrophage chemokine expression through oxidative stress-dependent and –independent mechanisms. J Immunol.

[9] Percario S, Moreira DR, Gomes BAQ, Ferreira MES, Gonçalves ACM, Laurindo PSOC (2012). Oxidative Stress in Malaria. Int J Mol Sci.

[10] Mau JL, Chao GR, Wu KT (2001). Antioxidant properties of methanolic extracts from several ear mushrooms. J Agric Food Chem.

[11] Gennari J, Veronesi R, Felippe J, Gennari MS, Percario S, Romaine CP, Keil CB, Rinker DL, Royse DJ (2004). Effect of *Agaricus sylvaticus* dietary supplementation on NK cell count in cancer patients. Science and cultivation of edible and medicinal fungi.

[12] Isah MB, Ibrahim MA (2014). The role of antioxidants treatment on the pathogenesis of malarial infections: a review. Parasitol Res.

[13] Liu F, Ooi VE, Chang ST (1997). Free radical scavenging activities of mushroom polysaccharide extracts. Life Sci.

[14] Percario S, Naufal AS, Gennari MS, Gennari JL (2009). Antioxidant activity of edible blushing wood mushroom, *Agaricus sylvaticus* Schaeff. (Agaricomycetideae) *in vitro*. Int J Med Mushr.

[15] Boulos M, Costa J, Tosta C (1993). Comprometimento pulmonar na malária. Rev Inst Med Trop São Paulo.

[16] Day N, Dondorp AM (2007). The management of patients with severe malaria. Am J Trop Med Hyg.

[17] Taylor WR, Canon V, White NJ (2006). Pulmonary manifestations of malaria: recognition and management. Treat Respir Med.

[18] Rojo-Marcos G, Cuadros-González J, Mesa-Latorre JM, Culebras-López AM, Pablo-Sánchez R (2008). Case report: acute respiratory distress syndrome in a case of *Plasmodium ovale* malaria. Am J Trop Med Hyg.

[19] Gachot B, Wolff M, Nissack G, Veber B, Vachon F (1995). Acute lung injury complicating imported *Plasmodium falciparum* malaria. Chest.

[20] Gillrie MR, Krishnegowda G, Lee K, Buret AG, Robbins SM, Looareesuwan S (2007). Src-family kinase–dependent disruption of endothelial barrier function by *Plasmodium falciparum* merozoite proteins. Blood.

[21] Pouvelle B, Matarazzo V, Jurzynski C, Nemeth J, Ramharter M, Rougon G (2007). Neural cell adhesion molecule, a new cytoadhesion receptor for *Plasmodium falciparum*-infected erythrocytes capable of aggregation. Infect Immun.

[22] Phiri H, Montgomery J, Molyneux M, Craig A (2009). Competitive endothelial adhesion between *Plasmodium falciparum* isolates under physiological flow conditions. Malar J.

[23] Suresh KP (2011). An overview of randomization techniques: an unbiased assessment of outcome in clinical research. J Human Reprod Sci.

[24] Sadavongvivad C, Aviado D (1969). Pathologic physiology and chemotherapy of *Plasmodium berghei*. VI. Mechanichal properties and histological features of the lung. Exp Parasitol.

[25] Aviado D, Camber PJ (1969). Pathologic physiology and chemotherapy of *Plasmodium berghei*. X. Pulmonary edema and naphthoquinones. Exp Parasitol.

[26] Weiss ML, Kubat K (1983). *Plasmodium berghei*: a mouse model for the “sudden death” and “malarial lung” syndromes. Exp Parasitol.

[27] Moore BR, Jago JD, Batty KT (2008). *Plasmodium berghei*: Parasite clearance after treatment with dihydroartemisinin in an asplenic murine malaria model. Exp Parasitol.

[28] Martins YC, Smith MJ, Pelajo-Machado M, Werneck GL, Lenzi HL, Daniel-Ribeiro CD (2009). Characterization of cerebral malaria in the outbred Swiss Webster mouse infected by *Plasmodium berghei* ANKA. Int J Path.

[29] Miller N, Rice-Evans C, Davies M, Gopinathan V, Milner A (1993). A novel method for measuring antioxidant capacity and its application to monitoring the antioxidant status in premature neonates. Clin Sci.

[30] Re R, Pellegrini R, Protegente A, Pannala A, Yang M, Rice-Evans C (1999). Antioxidant activity applying an improved ABTS radical cation decolorizaton assay. Free Rad Biol Med.

[31] Kohn HI, Liversedge M (1944). On a new aerobic metabolite whose production by brain is inhibited by apomorphine, emetine, ergotamine, epinephrine, and menadione. J Pharmacol Experimen Ther.

[32] Percario S, Vital A, Jablonka F (1994). Dosagem do malondialdeido. Newslab.

[33] May JM, Qu ZC, Xia L, Cobb CE (2000). Nitrite uptake and metabolism and oxidant stress in human erythrocytes. Am J Physiol Cell Physiol.

[34] Dell RB, Holleran S, Ramakrishnan R (2002). Sample size determination. ILAR J.

[35] Van den Steen PE, Geurts N, Deroost K, Van Aelst I, Verhenne S, Heremans H (2010). Immunopathology and dexamethasone therapy in a new model for malaria-associated acute respiratory distress syndrome. Am J Respir Crit Care Med.

[36] Berk M, Copolov D, Dean O, Lu K, Jeavons S, Schapkaitz I (2008). N-acetyl cysteine as a glutathione precursor for schizophrenia – a double-blind, randomized, placebo-controlled trial. Biol Psy.

[37] Carmeli C, Knyazeva MG, Cuénod M, Do KQ (2012). Glutathione precursor N-acetyl-cysteine modulates EEG synchronization in schizophrenia patients: a double-blind, randomized, placebo-controlled trial. PLoS ONE.

[38] Percario S, Odorizzi VF, Souza DRS, Pinhel MAS, Gennari JL, Gennari MS (2008). Edible mushroom *Agaricus sylvaticus* can prevent the onset of atheroma plaques in hypercholesterolemia rabbits. Cell Mol Biol.

[39] Fortes VC, Recôva VL, Melo AL, Novaes MRCG (2010). Life quality of postsurgical patients with colorectal cancer after supplemented diet with *Agaricus sylvaticus* fungus. Nutr Hosp.

[40] Fortes RC, Silva JRL, Novaes MRCG (2012). Anthropometric assessment of men with colorectal cancer after dietary supplement with *Agaricus sylvaticus* fungus. J Coloproctol.

[41] Orsine JVC, Brito LM, Silva RC, Almeida MFMS, Novaes MRCG (2013). Cytotoxicity of *Agaricus sylvaticus* in non-tumor cells (NTH/3T3) and tumor (OSCC-3) using tetrazolium (MTT) assay. Nutr Hosp.

[42] Figueira MS, Sa LA, Vasconcelos AS, Moreira DR, Laurindo PSOC, Ribeiro DRG (2014). Nutritional supplementation of mushroom *Agaricus sylvaticus* reversed oxidative stress in children with HIV. Can J Infec Dis Med Microbiol.

[43] Couper KN, Blount DG, Hafalla JC, Nico VR, Souza JB, Riley EM (2007). Macrophage-mediated but gamma interferon-independent innate immune responses control the primary wave of *Plasmodium yoelii* parasitemia. Infect Immun.

[44] Agbor-Enoh S, Seudieu C, Davidson E, Dritschilo A, Jung M (2009). Novel inhibitor of *Plasmodium* histone deacetylase that cures *P. berghei-*infected mice. Antimicr Agents Chemother.

[45] Epiphanio S, Campos M, Pamplona A, Carapau D, Pena AC, Ataíde R (2010). VEGF promotes malaria-associated acute lung injury in mice. PLoS Path.

[46] Saxena S, Kumar R, Madan T, Gupta V, Muralidhar K, Sarma PU (2005). Association of polymorphisms in pulmonary surfactant protein A1 and A2 genes with high-altitude pulmonary edema. Chest.

[47] Christofidou-Solomidou M, Scherpereel A, Wiewrodt R, Ng K, Sweitzer T, Arguiri E (2003). PECAM-directed delivery of catalase to endothelium protects against pulmonary vascular oxidative stress. Am J Physiol Lung Cell Mol Physiol.

[48] Oury TD, Schaefer LM, Fattman CL, Choi A, Weck KE, Watkins SC (2002). Depletion of pulmonary EC-SOD after exposure to hyperoxia. Am J Physiol Lung Cell Mol Physiol.

[49] Kinsella JP, Parker TA, Davis JM, Abman SH (2005). Superoxide dismutase improves gas exchange and pulmonary hemodynamics in premature lambs. Am J Respir Crit Care Med.

[50] Rahman I, MacNee W (2000). Oxidative stress and regulation of glutathione in lung inflammation. Eur Respir J.

[51] Hemmer CJ, Lehr HA, Westphal K, Unverricht M, Kratzius M, Reisinger EC (2005). *Plasmodium falciparum* malaria: reduction of endothelial cell apoptosis *in vitro*. Infect Immun.

[52] Trivedi V, Chand P, Srivastava K, Puri SK, Maulik PR, Bandyopadhyay U (2005). Clotrimazole inhibits hemoperoxidase of *Plasmodium falciparum* and induces oxidative stress. J Biol Chem.

[53] Keller CC, Kremsner PG, Hittner JB, Misukonis MA, Weinberg JB, Perkins DJ (2004). Elevated nitric oxide production in children with malarial anemia: hemozoin-induced nitric oxide synthase type 2 transcripts and nitric oxide in blood mononuclear cells. Infect Immun.

[54] Chen TH, Chang PC, Chang MC, Lin YF, Lee HM (2005). Chloroquine induces the expression of inducible nitric oxide synthase in C6 glioma cells. Pharmacol Res.

[55] Wang Q, Liu Y, Liu J, Chen G, Zheng W, Wang J (2009). *Plasmodium yoelii*: assessment of production and role of nitric oxide during the early stages of infection in susceptible and resistant mice. Exp Parasitol.

[56] Bichet C, Cornet S, Larcombe S, Sorci G (2012). Experimental inhibition of nitric oxide increases *Plasmodium relictum* (lineage SGS1) parasitemia. Exp Parasitol.

[57] Yeo TW, Lampah DA, Gitawati R, Tjitra E, Kenangalem E, McNeil YR (2007). Impaired nitric oxide bioavailability and L-arginine–reversible endothelial dysfunction in adults with falciparum malaria. JEM.

[58] Cabrales P, Zanini GM, Meays D, Frangos JA, Carvalho LJ (2011). Nitric oxide protection against murine cerebral malaria is associated with improved cerebral microcirculatory physiology. J Infect Dis.

[59] Janka JJ, Koita OA, Traoré B, Traoré JM, Mzayek F, Sachdev V (2010). Increased pulmonary pressures and myocardial wall stress in children with severe malaria. J Infect Dis.

[60] Bisseling TM, Roes ME, Raijmakers MTM, Steegers EAP, Peters WHM, Smits P (2004). N-acetylcysteine restores nitric oxide-mediated effects in the fetoplacental circulation of preeclamptic patients. Am J Obst Gynecol.

[61] Majano PL, Medina J, Zubía I, Sunyer L, Lara-Pezzi E, Maldonado-Rodríguez A (2004). N-acetyl-cysteine modulates inducible nitric oxide synthase gene expression in human hepatocytes. J Hepatol.

[62] González-Rubio S, Linares CI, Bello RI, González R, Ferrín G, Hidalgo AB (2010). Calcium-dependent nitric oxide production is involved in the cytoprotective properties of N-acetylcysteine in glycochenodeoxycholic acid-induced cell death in hepatocytes. Toxicol Ap Pharmacol.

[63] Straface E, Rivabene R, Masella R, Santulli M, Paganelli R, Malorni W (2002). Structural changes of the erythrocyte as a marker of non-insulin dependent diabetes: protective effects of N-acetylcysteine. Biochem Biophys Res Commun.

[64] Jiang B, Haverty M, Brecher P (1999). N-acetyl-L-cysteine enhances interleukin-1ß–induced nitric oxide synthase expression. Hypertension.

[65] Jiang B, Brecher P (2000). N-acetyl-L-cysteine potentiates interleukin-1ß induction of nitric oxide synthase: role of p44/42 mitogen-activated protein kinases. Hypertension.

[66] Fernhoff NB, Derbyshire ER, Marletta MA (2009). A nitric oxide/cysteine interaction mediates the activation of soluble guanylate cyclase. Proc Natl Acad Sci USA.

[67] Watt G, Jongsakul K, Ruangvirayuth R (2002). A pilot study of N-acetylcysteine as adjunctive therapy for severe malaria. Q J Med.

[68] Dhangadamajhi G, Mohapatra BN, Kar SK, Ranjit M (2009). Endothelial nitric oxide synthase gene polymorphisms and *Plasmodium falciparum* infection in Indian adults. Infect Immun.

[69] Zhu J, Krishnegowda G, Gowda DC (2005). Induction of proinflammatory responses in macrophages by the glycosylphosphatidylinositols of *Plasmodium falciparum*. J Biol Chem.

[70] Anstey NM, Handojo TH, Pain MC, Kenangalem E, Tjitra E, Price RN (2007). Lung injury in vivax malaria: pathophysiological evidence for pulmonary vascular sequestration and post treatment alveolar-capillary inflammation. J Infect Dis.

[71] Dimmeler S, Haendeler J, Nehls M, Zeiher AM (1997). Suppression of apoptosis by nitric oxide via inhibition of interleukin-1β–converting enzyme (ice)-like and cysteine protease protein (cpp)-32–like proteases. J Exp Med.

[72] Pino P, Taoufiq Z, Nicheu J, Vouldoukis I, Mazier D (2005). Blood–brain barrier breakdown during cerebral malaria: suicide or murder?. Thromb Haemost.

