# Supplementary material for: N-acetyl cysteine and mushroom Agaricus sylvaticus supplementation decreased parasitaemia and pulmonary oxidative stress in a mice model of malaria
Source: Malar J. 2015 May 15;14:202. doi: 10.1186/s12936-015-0717-0 (PMC4435846; doi:10.1186/s12936-015-0717-0)
Supplement: Supplementary file 1 — Medicinal properties of mushroom Agaricus sylvaticus . Presents the literature available about the medicinal properties of mushroom Agaricus sylvaticus and nutritional facts table of. [file 12936_2015_717_MOESM1_ESM.docx]

**Medicinal properties of mushroom *Agaricus sylvaticus***

Mushrooms have been used for medicinal purposes for thousands of years. Currently, there is a significant interest in the use of fungi as dietary supplements based on the theory that they increase immune function and promote health. The order Agaricales is one of the largest classes of fungi and contains a large number of important species that are used as nutritional supplement and therapeutic support (Taveira et al., 2008).

Pharmaceutical substances with powerful and unique health promoting properties have been recently isolated from medicinal mushrooms and distributed throughout the world. Many of these substances are pharmaceutical products, while others represent a new class of dietary supplements referred to as nutraceuticals (Wasser, 2002).

A few decades ago, epidemiologists studying the native population of the interior regions of the State of São Paulo in Brazil have noted that the rates of occurrence of some diseases in adults were extremely low, and realized it was related to frequent consumption of mushroom species of the genus Agaricus as part of the regular diet of the inhabitants of this area (Wasser, 2002).

Mushrooms of the genus Agaricus (*A. bisporus, A. blazei, A. sylvaticus* and others) have been extensively studied because of their medicinal properties, such as: antitumor (tumor growth inhibitor), antiangiogenic, hematological and immunological systems stimulator, antibacterial, nitric oxide-releaser, among others. Such properties have been attributed to the presence of specific molecules, among them glucans, ergosterol, some amino acids, such as arginine, among others (Wasser, 2002; Fortes et al., 2009; Ellertsen & Hetland, 2009; Smiderle et al., 2011).

Lately, medicinal and therapeutic use of mushrooms has been significantly increased. The species *Agaricus blazei* Murill (an edible Brazilian mushroom) presents particular immunomodulatory properties by means of bioactive compounds, promoting activation of both innate and adaptive immune response and complement system, and promoting the synthesis of pro- and anti-inflammatory cytokines. However, the precise mechanisms by which these properties occur are not yet fully elucidated, requiring randomized clinical studies with humans to apply the use of mushrooms in clinical practice (Hetland et al., 2008; Lima et al., 2011).

In addition to the immunomodulatory properties described, these biologically active substances present anti-inflammatory and antidiabetic effects, as well as stimulate hematopoiesis (Taveira et al., 2008).

Ellertsen & Hetland (2009) verified an anti-allergic activity of *Agaricus blazei* Murill extract in rats, with reduction of IgE-mediated allergy symptoms that can be reversed if previously established.

Liu et al. (2008) verified anti-tumor and anti-inflammatory effects and hepatocellular protection after supplementing *Agaricus brasiliensis* to rats, simultaneous to an increase in the number of helper T cells and NK cells.

The species *Agaricus sylvaticus*, popularly known as Sun Mushroom in Brazil, Himematsutake, Agarikusutake or Kawarihiratake in Japan and Ji Song Rong in China, was first described in Switzerland and has a wide geographic distribution, growing wild in Southern Brazil. It belongs to family Agaricaceae, and was first identified in the sixties in the region of Tapiraí (São Paulo, Brazil), having been subsequently taken to cultivation in Japan. Nowadays it represents an important commercial activity to this region, with several producers in Tapiraí surrounding cities, such as Pilar do Sul and Piedade (State of Sao Paulo, Brazil). Part of the crop is sold at regional markets, however, most of the production is exported to countries such as Japan, China and United States, representing an annual income of about US$ 8 million for the region (Firenzuoli et al., 2008; Percario et al., 2009).

Numerous antioxidant molecules were identified in extracts from this mushroom, such as vitamins E and D, various carbohydrates, bioflavonoids, and minerals (table 1). Rather than the antioxidant capacity from a single molecule, a synergistic effect is observed from the combination of antioxidant molecules from this unique mushroom, resulting in an extremely high antioxidant potential (Percario et al., 2008).

Additionally, both glucan and ergosterol exhibit antioxidant properties. Thus, it is likely that the medicinal properties displayed by *A. sylvaticus* arise mainly from the global capacity antioxidant, than one or other specific constituent (Percario et al., 2008).

Biochemical analyses carried out by the Department of Food Science at the University of Campinas (UNICAMP, Brazil), by Japan Food Research Laboratories and by the Food and Drug Administration (FDA) confirmed that this mushroom presents numerous antioxidant molecules, in addition to other important nutritional and therapeutic molecules (Table S1).

Unfortunately, to date only few pre-clinical or clinical studies about *A. sylvaticus* supplementation were performed. Fortes et al. (2008) evaluated the effects of dietary supplementation with the mushroom on fasting blood glucose levels of patients with colorectal cancer in post-surgical phase, comparing to placebo treated matched patients. They noted that these levels were significantly reduced as a consequence of supplementation, suggesting that this mushroom contains substances capable of reducing blood glucose, and beneficial effects in the regulation of carbohydrate metabolism.

Fortes et al. (2009) found that supplementation with *A. sylvaticus* promoted beneficial effects in hematological and immunological parameters in cancer patients.

Fortes et al. (2010) also reported an improvement in quality of life after the use of this supplementation in cancer patients, such as adherence to physical activity, improved mood, reduced on account of pain, insomnia reduction, appetite improvement, and reduced gastrointestinal changes, changes not observed in the placebo group.

Fortes and Novaes (2011) observed additional beneficial metabolic effects of this supplementation to colorectal cancer patients, such as normalization of several biochemical markers (glucose, total cholesterol, creatinine, alanine aminotransferase, aspartate aminotransferase, and IgA) in addition to better systolic and diastolic blood pressures.

Taveira et al. (2008) evaluated the effects of *A. sylvaticus* dietary supplementation on hematological and biochemical parameters of rats with cancer and observed that this supplementation is able to reduce anemia and improve biochemical parameters of these animals, without presenting adverse effects to blood cells in healthy animals.

Percário et al. (2008) investigated the potential benefit of antioxidant-rich mushroom *Agaricus sylvaticus* in the prevention of atherosclerosis and found that supplementation of this mushroom can prevent the development of atherosclerosis in rabbits.

Percário et al. (2009) also evaluated the *in vitro* antioxidant potential of *Agaricus sylvaticus* in different presentations and verified an antioxidant activity proportional to the concentration, with 100% of free radicals inhibition with extremely low quantities of the mushroom. These results suggest that *A. sylvaticus* is an important nutritional source of antioxidants due to its very high antioxidant capacity.

Gomes (2011) investigated the potential beneficial effect of *A. sylvaticus* supplementation on the oxidative changes induced in an experimental model of malaria and verified reduction in parasitemia and increased antioxidant capacity in lung and brain tissue of mice.

Silva (2011) investigated the effects of *A. sylvaticus* supplementation in lung tissues in a *Plasmodium berghei*-experimental model of malaria and verified reduction of parasitemia, as well as reduction of thiobarbituric acid reactive substances (an oxidative stress marker) and TNF-α, reducing the deleterious effects triggered by this cytokine such as NO synthesis, increased antioxidant capacity and increased production of Interferon γ (IFN-γ), cytokine required by host`s immune system to destroy the parasite.

Therefore, it is very likely that *A. sylvaticus* supplementation associated with conventional treatments may promote improved clinical condition in patients with various diseases. In the particular case of individuals suffering from malaria, the supplementation can strengthen host`s immune system at the same time it creates an unfavorable biochemical environment to the invasion of erythrocytes by the parasite, as well as to its own development.

REFERENCES

Ellertsen LK, Hetland G (2009) An extract of the medicinal mushroom *Agaricus blazei* Murill can protect against allergy. [Clin Mol Allergy](http://www.ncbi.nlm.nih.gov/pubmed/19416507) 7:6.

Firenzuoli F, Gori L, Lombardo G (2008) The Medicinal Mushroom *Agaricus blazei* Murrill: Review of Literature and Pharmaco-Toxicological Problems. eCAM 5(1):3–15.

Fortes RC, Novaes MRCG (2011) The effects of Agaricus sylvaticus fungi dietary supplementation on the metabolism and blood pressure of patients with colorectal cancer during post-surgical phase. Nutr Hospit 26(1):176-1861.

Fortes RC, Recôva VL, Melo AL, Novaes MRCG (2010) Life quality of postsurgical patients with colorectal cancer after supplemented diet with *Agaricus sylvaticus* fungus. Nutr Hosp 25(4):586-596.

Fortes RC, Novaes MRCG, Recova VL, Melo AL (2009) Immunological, hematological, and glycemia effects of dietary supplementation with *Agaricus sylvaticus* on patients’ colorectal cancer. Exp Biol Med 234:53–62.

Fortes RC, Recôva VL, Melo AL, Novaes MRCG (2008) Effects of Dietary Supplementation with Medicinal Fungus in Fasting Glycemia Levels of Patients with Colorectal Cancer: a Randomized, Double-blind, Placebo-controlled Clinical Study. Nutr Hosp 23(6):591-598.

Gomes BAQ (2011) Efeitos da suplementação com antioxidantes sobre as alterações oxidativas cerebral e pulmonar em malária murina. Master Thesis (Tropical Diseases), Federal University of Para, Belém, Para – Brazil.

[Hetland G](http://www.ncbi.nlm.nih.gov/pubmed?term=%22Hetland%20G%22%5BAuthor%5D), [Johnson E](http://www.ncbi.nlm.nih.gov/pubmed?term=%22Johnson%20E%22%5BAuthor%5D), [Lyberg T](http://www.ncbi.nlm.nih.gov/pubmed?term=%22Lyberg%20T%22%5BAuthor%5D), [Bernardshaw S](http://www.ncbi.nlm.nih.gov/pubmed?term=%22Bernardshaw%20S%22%5BAuthor%5D), [Tryggestad AM](http://www.ncbi.nlm.nih.gov/pubmed?term=%22Tryggestad%20AM%22%5BAuthor%5D), et al. (2008) Effects of the medicinal mushroom *Agaricus blazei* Murill on immunity, infection and cancer. [Scand J Immunol](http://www.ncbi.nlm.nih.gov/pubmed/18782264) 68(4):363-370.

[Lima CU](http://www.ncbi.nlm.nih.gov/pubmed?term=%22Lima%20CU%22%5BAuthor%5D)JO, [Cordova CO](http://www.ncbi.nlm.nih.gov/pubmed?term=%22Cordova%20CO%22%5BAuthor%5D)D, [Nóbrega OD](http://www.ncbi.nlm.nih.gov/pubmed?term=%22N%C3%B3brega%20Ode%20T%22%5BAuthor%5D), [Funghetto SS](http://www.ncbi.nlm.nih.gov/pubmed?term=%22Funghetto%20SS%22%5BAuthor%5D), [Karnikowski MG](http://www.ncbi.nlm.nih.gov/pubmed?term=%22Karnikowski%20MG%22%5BAuthor%5D)D (2011) Does the Agaricus blazei Murill mushroom have properties that affect the immune system? An integrative review. [J Med Food](http://www.ncbi.nlm.nih.gov/pubmed/21128829) 14(1-2):2-8.

[Liu Y](http://www.ncbi.nlm.nih.gov/pubmed?term=%22Liu%20Y%22%5BAuthor%5D), [Fukuwatari Y](http://www.ncbi.nlm.nih.gov/pubmed?term=%22Fukuwatari%20Y%22%5BAuthor%5D), [Okumura K](http://www.ncbi.nlm.nih.gov/pubmed?term=%22Okumura%20K%22%5BAuthor%5D), [Takeda K](http://www.ncbi.nlm.nih.gov/pubmed?term=%22Takeda%20K%22%5BAuthor%5D), [Ishibashi KI](http://www.ncbi.nlm.nih.gov/pubmed?term=%22Ishibashi%20KI%22%5BAuthor%5D), et al. (2008) Immunomodulating Activity of *Agaricus brasiliensis* KA21 in Mice and in Human Volunteers. [Evid Based Complement Alternat Med](http://www.ncbi.nlm.nih.gov/pubmed?term=Immunomodulating%20Activity%20of%20Agaricus%20brasiliensis%20KA21%20in%20Mice) 5(2):205-219.

Percario S, Naufal AS, Gennari MS, Gennari JL (2009) Antioxidant activity of edible blushing wood mushroom, *Agaricus sylvaticus* Schaeff. (Agaricomycetideae) *in vitro*. Int J Med Mushr 11(2):133-139.

Percario S, Odorizzi VF, Souza DRS, Pinhel MAS, Gennari JL, et al. (2008) Edible mushroom *Agaricus sylvaticus* can prevent the onset of atheroma plaques in hypercholesterolemia rabbits. Cell Mol Biol 54:1055-1061.

**Silva LFD (2011) Efeito da suplementação com antioxidantes sobre as alterações oxidativas e produção de interferon gama e fator de necrose tumoral alfa em tecido pulmonar de camundongos infectados por *Plasmodium berghei*. Master Thesis (Biology of Infectious and Parasitary Agents), Federal University of Pará, Belém, Para – Brazil.**

Smiderle FR, Ruthes AC, Van Arke lJ, Chanput W, Iacomini M, et al. (2011) Polysaccharides from *Agaricus bisporus* and *Agaricus brasiliensis* show similarities in their structures and their immunomodulatory effects on human monocytic THP-1 cells. BMC Complement Altern Med 11(1):58.

Taveira VC, Novaes MRCG, Reis MA, Silva MF (2008) Hematologic and Metabolic Effects of Dietary Supplementation with *Agaricus sylvaticus* Fungi on Rats Bearing Solid Walker 256 Tumor. Exp Biol Med 233(11):1341-1347.

Wasser SP (2002) Review of medicinal mushrooms advances: good news from old allies. Herbal Gram 56:28–33.

**Nutritional Facts of *Agaricus sylvaticus***

| **Nutrient** | **Amount** | **Nutrient** | **Amount** |
| --- | --- | --- | --- |
| Proteins | 39,4 mg/100 g | Arginine | 1,71 g/100 g |
| Fats | 3,0 g/100 g | Lysine | 1,55 g/100 g |
| Carbohydrates | 45,6 g/100 g | Histidine | 0,62 g/100 g |
| Sodium | 4,2 mg/100 g | Phenylalanine | 1,11 g/100 g |
| Iron | 21,2 mg/100 g | Tyrosine | 0,83 g/100 g |
| Calcium | 35,7 mg/100 g | Leucine | 1,72 g/100 g |
| Potassium | 3,15 g/100 g | Isoleucine | 1,10 g/100 g |
| Magnesium | 100 mg/100 g | Methionine | 0,39 g/100 g |
| Copper | 8,24 mg/100 g | Valine | 1,28 g/100 g |
| Zinc | 6,61 mg/100 g | Alanine | 1,75 g/100 g |
| Manganese | 0.65 mg/100 g | Glycine | 1,25 g/100 g |
| Selenium | 36 µg/100 g | Proline | 1,26 g/100 g |
| Thiamine (B1) | 1,21 mg/100 g | Glutamic acid | 5,73 g/100 g |
| Riboflavin (B2) | 3,41 mg/100 g | Serina | 1,20 g/100 g |
| Vitamin B6 | 0,83 mg/100 g | Threonine | 1,21 g/100 g |
| Vitamin B12 | 0,17 µg/100 g | Aspartic acid | 2,35 g/100 g |
| Vitamin C | 56 mg/100 g | Tryptophan | 0,43 g/100 g |
| Calciferol | 5,8 µg/100 g | Cistine | 0,36 g/100 g |
| Folic acid | 0,36 mg/100 g | Sugar Free | 282 mg/g |
| Pantothenic acid | 39,4 mg/100 g | Phenols | 0,10 mg/g |
| Inositol | 201 mg/100 g | Beta d glucan | 126,98 mg/g |
| Niacin | 39,9 mg/100 g | Isoflavones (genistein) | 0,875 mg/g |
