# Supplementary material for: N-acetyl cysteine and mushroom Agaricus sylvaticus supplementation decreased parasitaemia and pulmonary oxidative stress in a mice model of malaria
Source: Malar J. 2015 May 15;14:202. doi: 10.1186/s12936-015-0717-0 (PMC4435846; doi:10.1186/s12936-015-0717-0)
Supplement: Supplementary file 2 — Number of red blood cells to be counted. Contains a board of number of red blood cells to be counted from an initial parasitemia estimative. [file 12936_2015_717_MOESM2_ESM.docx]

**Number of red blood cells to be counted from an initial parasitemia estimative**

| **Parasitemia (%)** | **Erythrocytes counted** |
| --- | --- |
| 0 | 10,000 |
| <6 | 5,000 |
| 6-10 | 2,000 |
| 11 20 | 1,000 |
| >20 | 300 |
