# Supplementary material for: N-acetyl cysteine and mushroom Agaricus sylvaticus supplementation decreased parasitaemia and pulmonary oxidative stress in a mice model of malaria
Source: Malar J. 2015 May 15;14:202. doi: 10.1186/s12936-015-0717-0 (PMC4435846; doi:10.1186/s12936-015-0717-0)
Supplement: Supplementary file 3 — Pulmonary Nitrites and Nitrates (NN) of Plasmodium berghei- infected mice in groups supplemented with N-Acetyl cysteine (NAC) or Agaricus sylvaticus (AS) and control groups, accordingly to duration of infection. Presents mean ± standard deviation values of nitrites and nitrates for each group. [file 12936_2015_717_MOESM3_ESM.docx]

**Table – Pulmonary Nitrites and Nitrates (NN) of *Plasmodium* *berghei-*infected mice in groups supplemented with N-Acetyl cysteine (NAC) or *Agaricus sylvaticus* (AS) and control groups, accordingly to duration of infection**

| **Group** | **NN (mmol/L)** | | | | | ***p**** |
| --- | --- | --- | --- | --- | --- | --- |
|  | **1 day** | **3 days** | **5 days** | **7 days** | **10 days** |  |
| **PC** | 2.9±1.0 | 1.9±1.5 | 4.7±3.5^#^ | 2.4±1.7^#^ | 4.4±1.9^#^ | 0.0783 |
| **NC** | 0.9±0.2 | 0.6±0.1 | 0.8±0.4 | 0.7±0.1 | 0.9±0.3 | 0.7039 |
| **NAC** | 2.5±2.5 | 3.0±1.8^€^ | 8.9±4.0^€^ | 7.4±1.5^€§^ | 13.3±9.0^€§^ | 0.0025 |
| **AS** | 1.2±0.5 | 1.0±0.4 | 0.7±0.4 | 0.6±0.2 | 0.6±0.2 | 0.0171 |
| Values presented as mean ± standard deviation. PC= animals infected with *P. berghei*, but not supplemented; NC= animals not infected and not supplemented. * t Student 10 days *versus* 1 day of infection. ^€^ p<0.01xAS or NC; ^#^ p<0.05xAS or NC; ^§^ p<0.01xPC. Group size: NC (1day N=9*/10; 3days N=8*/10; 5days N=9*/10; 7days N=8*/10; 10days N=9*/10); PC (1day N=9*/10; 3days N=10/10; 5days N=9^†^/10; 7days N=7^†^/10; 10days N=6^†^/10); NAC (1day N=10/10; 3days N=8*/10; 5days N=9*/10; 7days N=6^†*^/10; 10days N=6^†^/10); AS (1day N=10/10; 3days N=10/10; 5days N=9*/10; 7days N=8^†^/10; 10days N=6^†^/10). Discrepancies in group size were due to dead animals (^†^) or outliers (*). | | | | | | |
