# Supplementary material for: N-acetyl cysteine and mushroom Agaricus sylvaticus supplementation decreased parasitaemia and pulmonary oxidative stress in a mice model of malaria
Source: Malar J. 2015 May 15;14:202. doi: 10.1186/s12936-015-0717-0 (PMC4435846; doi:10.1186/s12936-015-0717-0)
Supplement: Supplementary file 4 — Correlation between Nitrites and nitrates (NN) and Thiobarbituric acid reactive substances (TBARS) in lung tissue of mice. Present the correlation study charts of NN versus TBARS and consolidated charts of the variation of the average values of NN and TBARS in lung tissue of mice with the time of infection for each group. [file 12936_2015_717_MOESM4_ESM.docx]

**Ahe**

**B**

**C**

**D**

**Figure 1S. Correlation between Nitrites and nitrates (NN) and Thiobarbituric acid reactive substances (TBARS) in lung tissue of mice.** **A**= all animals of all groups and subgroups simultaneously; **B**= only animals of subgroups of the positive control group, in which the animals were infected with *Plasmodium* *berghei*; **C**= only animals of the subgroups of group N-acetyl cysteine (NAC), in which the animals were infected with *P. berghei* and supplemented with NAC; **D**= only animals of subgroups of *Agaricus* *sylvaticus* (AS), in which the animals were infected with *P*. *berghei* and supplemented with AS.

**B**

**C**

**Figure 2S. Variation of the average values of Nitrites and nitrates (NN) and Thiobarbituric acid reactive substances (TBARS) in lung tissue of mice with the time of infection.** **A**= positive control group, in which the animals were infected with *Plasmodium* *berghei*; **B**= group N-acetyl cysteine (NAC), in which the animals were infected with *P. berghei* and supplemented with NAC; **C**= *Agaricus* *sylvaticus* (AS), in which the animals were infected with *P. berghei* and supplemented with AS.
