# Supplementary material for: N-acetyl cysteine and mushroom Agaricus sylvaticus supplementation decreased parasitaemia and pulmonary oxidative stress in a mice model of malaria
Source: Malar J. 2015 May 15;14:202. doi: 10.1186/s12936-015-0717-0 (PMC4435846; doi:10.1186/s12936-015-0717-0)
Supplement: Supplementary file 8 — Correlation between Parasitemia and Thiobarbituric acid reactive substances (TBARS) in lung tissue of mice. Present the correlation study charts of Parasitemia versus TBARS and consolidated charts of the variation of the average values of Parasitemia and TBARS in lung tissue of mice with the time of infection for each group. [file 12936_2015_717_MOESM8_ESM.docx]

**Figure 9S. Correlation between Parasitemia and Thiobarbituric acid reactive substances (TBARS) in lung tissue of mice.** **A**= all animals of all groups and subgroups simultaneously; **B**= only animals of subgroups of the positive control group, in which the animals were infected with *Plasmodium* *berghei*; **C**= only animals of the subgroups of group N-acetyl cysteine (NAC), in which the animals were infected with *P. berghei* and supplemented with NAC; **D**= only animals of subgroups of *Agaricus* *sylvaticus* (AS), in which the animals were infected with *P*. *berghei* and supplemented with AS.

**Figure 10S. Variation of the average values of Parasitemia and Thiobarbituric acid reactive substances (TBARS) in lung tissue of mice with the time of infection**. **A**= positive control group, in which the animals were infected with *Plasmodium* *berghei*; **B**= group N-acetyl cysteine (NAC), in which the animals were infected with *P. berghei* and supplemented with NAC; **C**= *Agaricus* *sylvaticus* (AS), in which the animals were infected with *P. berghei* and supplemented with AS.
