# Supplementary material for: N-acetyl cysteine and mushroom Agaricus sylvaticus supplementation decreased parasitaemia and pulmonary oxidative stress in a mice model of malaria
Source: Malar J. 2015 May 15;14:202. doi: 10.1186/s12936-015-0717-0 (PMC4435846; doi:10.1186/s12936-015-0717-0)
Supplement: Supplementary file 9 — Survival rate of Plasmodium berghei- infected mice in groups supplemented with N-Acetyl cysteine (NAC) or Agaricus sylvaticus (AS) and control groups, accordingly to duration of infection. Presents the survival rate chart for all groups. [file 12936_2015_717_MOESM9_ESM.docx]

**Survival in *Plasmodium berghei*-infected mice and supplemented with N-acetyl cysteine (NAC) or *Agaricus sylvaticus* (AS)**

PC= animals infected with *P. berghei*, but not supplemented; NC = animals not infected and not supplemented.
